# *Leishmania infantum* in Tigers and Sand Flies from a Leishmaniasis-Endemic Area, Southern Italy

**DOI:** 10.3201/eid2606.191668

**Published:** 2020-06

**Authors:** Roberta Iatta, Andrea Zatelli, Pietro Laricchiuta, Matteo Legrottaglie, David Modry, Filipe Dantas-Torres, Domenico Otranto

**Affiliations:** University of Bari, Bari, Italy (R. Iatta, A. Zatelli, F. Dantas-Torres, D. Modry, D. Otranto);; Zoo Safari di Fasano, Brindisi, Italy (P. Laricchiuta, M. Legrottaglie);; University of Veterinary and Pharmaceutical Sciences, Brno, Czech Republic (D. Modry);; Czech Academy of Sciences, Ceske Budejovice, Czech Republic (D. Modry);; Masaryk University, Brno (D. Modry);; Fundação Oswaldo Cruz (Fiocruz), Recife, Brazil (F. Dantas-Torres);; Bu-Ali Sina University, Hamedan, Iran (D. Otranto).

**Keywords:** Leishmania infantum, infection, IFAT, real-time PCR, sand flies, blood meal preferences, tigers, protozoa, parasites, leishmaniasis, Italy

## Abstract

We detected *Leishmania infantum* infection in 45% of tigers and 5.3% of sand flies tested at a zoo in southern Italy in 2019. These infections in tigers and the abundance of *Phlebotomus perniciosus* sand flies represent a potential risk to other animals and humans living in or visiting the zoo.

Visceral leishmaniasis, caused by infection with *Leishmania infantum* protozoa, is listed among the most neglected tropical diseases, affecting thousands of persons, most of whom are among the world’s most vulnerable populations (*1*). The disease is associated with the presence of phlebotomine sand fly vectors; domestic dogs typically act as reservoirs. Among felids, domestic cats have recently gained prominence as putative reservoirs of *L. infantum* (*2*), whereas cases of infection in other felids have been reported occasionally (*3*–*5*). 

In February 2019, a tiger (index case), born and raised in a zoologic park in southern Italy, had a nonhealing laceration that tested positive for *L. infantum* DNA on a skin punch biopsy. Because tigers are considered an endangered species, the presence of an active *L. infantum* transmission focus in a facility visited by thousands of visitors each year deserves attention. Therefore, we conducted an epidemiologic study to investigate the prevalence of *L. infantum* infection in the local tiger and sand fly populations, along with the sand flies’ host blood-feeding preferences.

## The Study

During March–June 2019, we tested 20 tigers born at the zoologic park (Safari Park, Apulia region, Brindisi Province, southern Italy) and living in an open enclosure for *L. infantum* infection. We smeared lymph node aspirates on slides for the cytologic examination; we also cultured and processed these specimens, along with whole blood, skin punch biopsy, and conjunctival, nasal and oral swab specimens, for the detection of *L. infantum* DNA by quantitative PCR (qPCR) (*6*). We tested for feline leukemia virus (FeLV) and feline immunodeficiency virus (FIV) by using proviral DNA from blood, as described previously (*7*). We detected *L. infantum* antibodies by using an immunofluorescence antibody test (IFAT), as described previously in a study in cats (*2*). During May–November 2019, we collected sand flies in the tigers’ enclosure biweekly by using sticky traps and light traps and identified each specimen by using morphologic keys. We performed conventional PCR for blood-meal identification in sand flies by using primers cyto 1 (5′-CCATCAAACATCTCAGCATGAAA-3′) and T2893R (5′-GTTGGCGGGGATGTAGTTATC-3′), which target the mitochondrial cytochrome b. The protocol of this study was approved by the ethics committee of the Department of Veterinary Medicine at the University of Bari (Bari, Italy).

Tigers enrolled in the study ranged in age from 6 months to 11 years and weighed 70–220 kg (Table 1); all were apparently healthy or had unrelated conditions, except for 1 (index case), which had a large nonhealing laceration extending from the left loin region to the left thoracic region (Figure). Overall, 9 (45%) of the 20 tigers tested positive for *L. infantum* by IFAT, 5 (25%) tested positive by qPCR, and 5 (25%) tested positive by both methods (Table 1). The tigers were positive by qPCR on lymph node aspirates and skin punch biopsy. None of the conjunctival swab specimens tested positive. We did not detect *L. infantum* cytology or culture of lymph node aspirates in any of the tigers. All tigers were negative for FeLV and FIV.

**Table 1:** Serologic and molecular results for *Leishmania infantum* in 20 tigers, southern Italy*

Month of sample collection	Case no.	Age, y/sex	Weight, kg	IFAT/Ab titer	qPCR results					
					PB	LN	CS	NS	OS	SPB
March	1	7/F	150	1:160	–	+	–	–	–	+
April	2	6/M	160	1:80	–	NT	–	–	–	–
April	3	7/M	220	1:80	–	–	–	–	–	–
April	4	8/M	210	1:80	–	NT	–	+	+	–
April	5	7/M	220	–	–	–	–	–	–	–
April	6	9/F	150	–	–	–	–	–	–	–
May	7	2/F	135	1:640	–	+	–	+	–	+
May	8	2/M	180	–	–	NT	–	–	–	–
May	9	2/F	150	1:40	+	+	–	–	–	+
May	10	2/M	170	1:40	+	+	–	–	+	+
May	11	8/F	190	1:40	–	–	–	–	–	–
May	12	6/M	220	–	–	–	–	–	–	–
May	13	7/F	160	–	–	–	–	–	–	–
May	14	11/F	120	–	–	–	–	–	–	–
June	15	1/M	130	–	–	–	–	–	–	–
June	16	1/F	110	–	–	–	–	–	–	–
June	17	7/F	150	1:80	–	–	–	–	–	–
June	18	0.5/F	70	–	–	–	–	–	–	–
June	19	0.5/F	70	–	–	–	–	–	–	–
June	20	0.5/F	70	–	–	–	–	–	–	–

*Ab, antibody; CS, conjunctival swab; IFAT, immunofluorescence antibody test; LN, lymph node; NS, nasal swab; NT, not tested; OS, oral swab; PB, peripheral blood; qPCR, quantitative PCR; SPB, skin punch biopsy; –, negative; +, positive.

**Figure:**
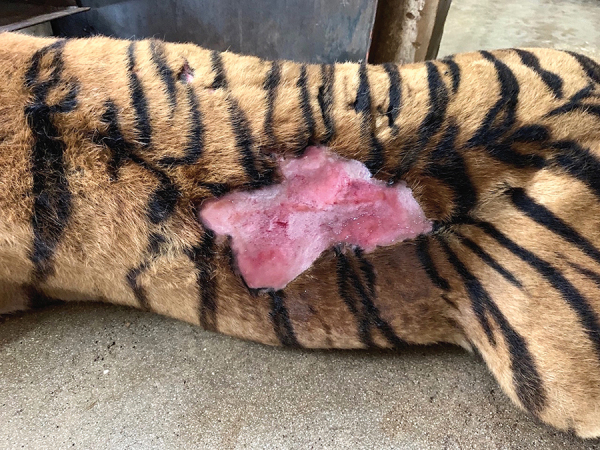
Large nonhealing laceration, attributable to *Leishmania infantum* infection, extending from the left loin region to the left thoracic region of a tiger, southern Italy.

During May–November 2019, we collected a total of 580 sand flies. The most abundant species was *Phlebotomus perniciosus* (n = 491), followed by *Sergentomyia minuta* (n = 69) and *P. neglectus* (n = 20). Of the 190 females collected, 151 (26%) were *P. perniciosus*, 4 (<1%) were *P. neglectus*, and 35 (6%) were *S. minuta*. Specimens for 8 (5.3%) *P. perniciosus* sand flies and 1 (2.9%) *S. minuta* sand fly tested positive for *L. infantum* DNA. Of the 190 females examined, 63 (33.1%) *P. perniciosus*, 3 (1.6%) *P. neglectus*, and 2 (1.1.%) *S. minuta* sand flies tested positive for tiger DNA (Table 2); we detected no other mammalian DNA (e.g., from cats, dogs, rats, or humans) in blood-fed or -unfed specimens. Consensus sequences of the vertebrate host mitochondrial cytochrome b from all female sand flies (positive specimens) displayed 100% identity to the nucleotide sequences of *Panthera tigris* available in the GenBank database (accession nos. MH124112 and KC879295). 

**Table 2:** Number of phlebotomine sand fly species by sex, positivity for *Leishmania infantum* by quantitative PCR, and blood meal on tigers, southern Italy*

Sand fly species	Sex		Total	No. (%) positive for *L. infantum*	Blood meal source	
	F	M			No. positive	No. positive/engorged
*Phlebotomus perniciosus*	151	340	491	8/151 (5.3)	63/151	48/63
*Sergentomyia minuta*	35	34	69	1/35 (2.9)	2/35	–
*Phlebotomus neglectus*	4	16	20	–	3/4	–
Total	190	390	580	–	68/190	48/63
*–, no result.						

## Conclusions

The high prevalence (45%) of *L. infantum* infection recorded indicates that tigers living in the zoologic park are highly exposed to sand flies and thus have a high risk for acquiring the parasite. The finding of engorged sand flies that fed on tigers and were also positive for *L. infantum* suggest that tigers could be an alternative host of this parasite; however, the possibility that *L. infantum*–positive sand flies had acquired the infection from another host, before feeding on tigers, cannot be ruled out.

Although *Leishmania* spp. infection has been scantly described in wild felids (*3*–*5*), the diagnosis of this parasitic infection should also be considered while screening these animals for pathogens potentially impairing their health and welfare. No information is available on the immune response against *L. infantum* infection in tigers, and serologic tests have not been validated for this host, but one could reasonably suspect that their antibody production would follow a pattern similar to that occurring in cats. Nonetheless, the absence of *L. infantum* DNA in tigers that were positive for *L. infantum* antibodies (4/9 tigers [44.4%]) could be expected, given that this lack of correlation between molecular and serologic positivity has also been observed in cats (*2*), indicating that the diagnosis of the infection in these animals might be a difficult task, as it is in cats. The detection of *L. infantum* DNA in the lymph node aspirate and skin biopsy suggests that these tissues are more suitable than blood for the diagnosis of this infection, as previously reported in dogs and cats (*8*,*9*). Otherwise, the conjunctival swab seems to be not as good a sample for this purpose in tigers. Unlike some studies with cats (*2*), no correlation between *L. infantum* infection and FIV, FELV, or both FIV and FELV infection has been observed in the tigers in our study.

The predominance of *P. perniciosus* sand flies, along with their positivity for *L. infantum* DNA already recorded in southern Italy (*10*,*11*), is somewhat expected, given that this sand fly species is recognized as the main vector for *L. infantum* in different foci of visceral leishmaniasis in Italy (*12*). The high proportion of *L. infantum*–infected sand flies suggests that the risk for parasite transmission in this environment should be considered. Furthermore, the detection of *L. infantum* DNA in *S. minuta* sand flies has already been reported in southern Italy (4.2%) and Portugal (4%) (*11*,*13*). In addition, although consideration of the role played by *S. minuta* (the proven vector of *L. tarentolae*) in the circulation of *Leishmania* spp. of zoonotic concern has been raised (*11*,*14*), further studies are necessary to fully assess its vector role.

*P. perniciosus* sand flies frequently feed on tigers, because dogs are not allowed to roam in the zoo, the role of tigers as local reservoir hosts needs to be ascertained. Because *P. perniciosus* sand flies feed on a wide range of domestic and wild animals, and because *L. infantum* might infect the sand flies after taking a blood meal from infected felids (*15*), the role of tigers in the transmission cycle of *L. infantum* is probable.

In summary, *L. infantum* infection should be included in the differential diagnosis of infectious diseases in tigers in areas where visceral leishmaniasis is endemic. The role of tigers as sentinels for *L. infantum*, the occurrence of *P. perniciosus* sand flies infected by the protozoan, and its abundance in the study area might represent an eminent risk for animals and humans living in or visiting the zoo. Therefore, prevention measures are needed for providing protection against *L. infantum* infection in these animals and for controlling sand flies.
